# Recent technological developments: *in situ *histopathological interrogation of surgical tissues and resection margins

**DOI:** 10.1186/1746-160X-3-13

**Published:** 2007-03-01

**Authors:** Tahwinder Upile, Cyril Fisher, Waseem Jerjes, Mohammed El Maaytah, Sandeep Singh, Holger Sudhoff, Adam Searle, Daniel Archer, Leslie Michaels, Colin Hopper, Peter Rhys-Evans, David Howard, Anthony Wright

**Affiliations:** 1 Department of Head & Neck Surgery, the Royal Marsden Hospital, London, UK; 2 Department of Histopathology, the Royal Marsden Hospital, London, UK; 3 Unit of Oral & Maxillofacial Surgery, Division of Maxillofacial, Diagnostic, Medical and Surgical Sciences, Eastman Dental Institute & University College London, London, UK; 4 Department of Plastic and Reconstructive Surgery, the Royal Marsden Hospital, London, UK; 5 Department of Head & Neck Surgery, the Royal Marsden Hospital, London, UK; 6 Department of Head & Neck Surgery, the Professorial Unit, the Royal National Throat, Nose and Ear Hospital, London, UK; 7 Department of Oral & Maxillofacial Surgery/Head & Neck Unit, University College London Hospitals, London, UK; 8 National Medical Laser Centre, Department of Surgery, Royal Free & University College Medical School, London, UK

## Abstract

**Objectives:**

The tumour margin is an important surgical concept significantly affecting patient morbidity and mortality. We aimed in this prospective study to apply the microendoscope on tissue margins from patients undergoing surgery for oral cancer *in vivo *and *ex vivo *and compare it to the gold standard "paraffin wax", inter-observer agreement was measured; also to present the surgical pathologist with a practical guide to the every day use of the microendoscope both in the clinical and surgical fields.

**Materials and methods:**

Forty patients undergoing resection of oral squamous cell carcinoma were recruited. The surgical margin was first marked by the operator followed by microendoscopic assessment. Biopsies were taken from areas suggestive of close or positive margins after microendoscopic examination. These histological samples were later scrutinized formally and the resection margins revisited accordingly when necessary.

**Results:**

Using the microendoscope we report our experience in the determination of surgical margins at operation and later comparison with frozen section and paraffin section margins "gold standard". We were able to obtain a sensitivity of 95% and a specificity of 90%. Inter-observer Kappa scores comparing the microendoscope with formal histological analysis of normal and abnormal mucosa were 0.85.

**Conclusion:**

The advantage of this technique is that a large area of mucosa can be sampled and any histomorphological changes can be visualized in real time allowing the operator to make important informed decisions with regards the intra-operative resection margin at the time of the surgery.

## Background

The mucosal surface of the upper aero-digestive tract is bathed in a 'milieu' including toxins which can give rise to disease when the host repair processes are overcome. These disease processes may be discrete or multi-focal and can occur anywhere within the mucosal blanket. The usual epithelial response to this chronic injury is squamous metaplasia and hyperplasia, in the case of columnar epithelium. Epithelial hyperplasia can be manifest as keratosis clinically recognized as white patches (leukoplakia). Further disruption of this already unstable mucosa can engender carcinogenic changes leading to the development of squamous cell carcinoma, which again may be multi-focal and difficult to differentiate from the surrounding unstable mucosa by simple observation. Several areas of mucosa can be involved in this pathway to malignancy; hence any examination of the mucosa must be detailed and comprehensive in-order not to miss subtle lesions.

The diagnosis of dysplastic pre-malignant lesions cannot solely be based on clinical findings. Histological evaluation of a representative specimen is necessary. Dysplasia and carcinoma *in situ *herald invasive oral cancer [1], but carcinomas can occur in areas with no previous signs of dysplasia. This may be because of the rapid emergence of invasive cancer, or it may be that earlier biopsies were taken from unrepresentative sites of the lesion or before morphological changes could be detected. Furthermore, the grading of dysplasia also suffers from inter-observer variability [2].

There is no reliable method applicable to the upper aero-digestive tract that can replace a biopsy for a definitive diagnosis of malignancy but some may be used as a supplement. Exfoliative cytology carries the risk of false positive or negative results; a biopsy is still necessary for final diagnosis. Vital dyes have been used to identify a suitable site for biopsy, but literature has shown that the risk of false positive staining may be as high as 30% in the oral cavity [3]. This is mainly caused by the enhanced staining of the hyperplastic edges of ulceration and filliform papillae of the tongue.

Histological assessment of a tissue sample is regarded as the most reliable criterion for correct diagnosis; accordingly, the specimen must be taken from the most representative area. In cases involving the uterine cervix (which can also undergo squamous metaplastic changes), microcolpohysteroscopy and colposcopy are used to examine the mucosa. This consists of mainly of assessing the vascular pattern, inter-capillary distance, surface contour, colour, tone, and clarity of demarcation. The accuracy of colposcopy for the detection of mucosal changes is between 70–98% [4]. Gynther et al. [5] showed that direct microscopy of pre-stained oral mucosal lesions (with magnifications of up to ×8, ×12 and ×20) offered an advantage in selecting more representative sites for biopsy than routine clinical examination alone.

The next step is to obtain accurate microscopic assessment at the time of clinical examination, to inform the surgical decision as to the nature of the cellular characteristics of the mucosal lesion and its edge thereby aiding definitive biopsy yield and excision margin.

We believe this can be achieved by the use of the microendoscope. Microendoscopy was first popularized by Hamou in 1979 as a technique for the study of the epithelial cells of the uterus. The endoscope was later modified for use in the upper aero-digestive tract by Andreas [6,7]. However, it has continued to be regarded as an "orphan technology awaiting an application".

Microendoscopy allows *in vivo *examination of the epithelium. The endoscope allows the monitoring of the whole mucosal surface both normal and pathological, and allows the detection of patterns specific for pathology e.g. inflammation, metaplasia, dysplasia, and malignancy.

The use of the endoscope gives one immediate gratification at examination and can be used to guide further surgery, biopsy or simple surveillance of large areas of suspect mucosa. The microendoscope enables "on table" analysis to inform one's choices of further surgery even when frozen section biopsy is not available.

The aim of this prospective study was to apply the microendoscope on tissue margins from patients undergoing surgery for oral cancer *in vivo *and *ex vivo *and compare it to the gold standard "paraffin wax" histopathological analysis, inter-observer agreement was measured. We hope to present the surgical pathologist with a practical guide to the every day use of the microendoscope both in the clinical and surgical fields.

## Materials and methods

Forty consecutive patients (mean age 54 year, 28 males and 12 females) undergoing resection of early (T stage I/II) oral squamous cell carcinoma at the department of Head & Neck Surgery, University College London Hospitals, were recruited for this trial. The trial protocol was approved by the local committee of the ethics for human research.

An information sheet explaining the aim of our study in simple non-scientific terms was given to each patient who was then asked to sign a consent form prior to surgery.

**Figure 1:**
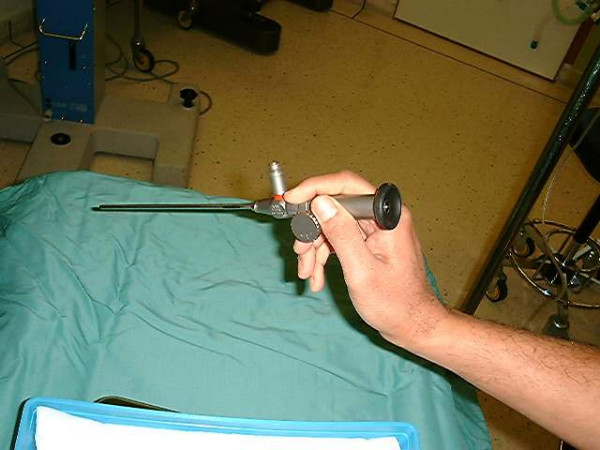
An operating Storz Hopkins II auto-clavable microendoscope capable of magnifications 0–150×. The rod can be angulated 30° or straight 0° with the cylinder of 4 mm in diameter allowing ready access to the oral and nasal, pharyngeal cavities as well as the overlying skin.

The operating Storz Hopkins II auto-clavable microendoscope (Figure 1) is attached via an adapter to a camera system (3 chips Olympus/Sony) linked via a video recorder with outputs to a monitor and photo-printer. Video encoding was done by using a Xenon-light source (Karl Storz 300 attached via a Storz fibre optic cable), a 3 CCD camera (Karl Storz Tricam) and a Sony DV Cam system (DSR-20P). The DV Cam recording enabled high resolution playback. Static documentation was performed in the form of simple photography (Sony printer/dpi) as well as dynamic (in the form of a video-clip, stored in a DV magnetic tape).

High quality photographic images can also be extracted from the edited video; this enabled a documented comparison of normal and abnormal epithelial areas as well as allowing surveillance of unstable mucosa over time.

The microendoscope has a fitted rotating screw which allows magnification to be changed from 0× to 60× to 150×. Minor movements permit focussing and de-focussing at specific depths of field. The generalised area would be surveyed at 0× magnification, until an area of interest was found; this would then be examined at 60× and then 150×. Examination would always proceed from an area of normality to abnormality; the whole of the surface of the lesion would be reviewed to determine any heterogeneity. The margin of the lesion would then be delineated.

The endoscope is available in different sizes which is can be used for different anatomical regions. The longer larger endoscopes (diameter 5.5 mm, length 23 cm) are more appropriate for oro-laryngeal lesions. Whilst Shorter microendoscopes (diameter 4 mm, length 18 cm) can be used for oro-nasal lesions.

The angulations of the endoscope also varied. The 0° endoscope is useful for medially placed lesions or those that could be directly approached and to which the tip may be applied with occlusive contact, e.g. floor of mouth, tongue, inferior turbinate, vocal folds...etc. The 30° forward oblique endoscope has better utility in more laterally placed pathology to obtain occlusive contact for subsequent focusing e.g. retro-molar trigone, lateral border of the tongue, buccal sulcus, nasal cavity...etc.

**Figure 2:**
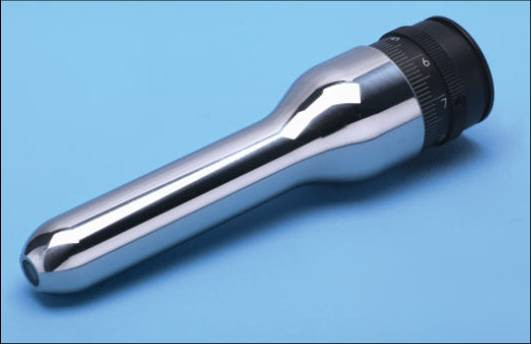
An auto-clavable "Cy-scope" microendoscope (P.W. Allen), allowing magnifications of 0–1000× *in situ*. The scope can be used on the skin and in the oral cavity and at operation to assess tissues and tumour margins.

Recently we have used a newly developed "Cy-scope" microendoscope (P.W. Allen) which has given improved magnifications of up to 300 times (*in vivo*) and 1000 times (*in vitro*) by modifying their optical systems (Figure 2). The methodology for microendoscopy remains the same.

**Figure 3:**
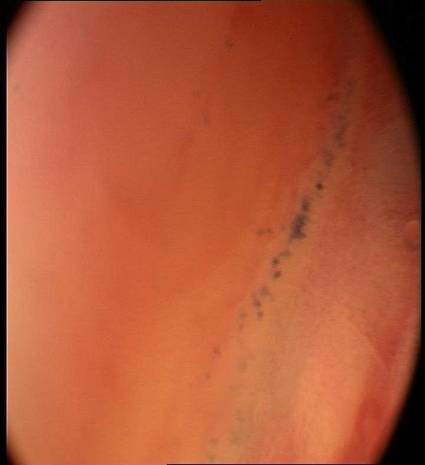
Initial microendoscopic examination (x60) of buccal mucosa stained with methylene blue showing an area of high nuclear density visible as a streak. This is because at the microscopic level the epithelial surfaces are ruffled or concertinaed which distorts the image viewed and causes the impression of a high nuclear density per image field.

**Figure 4:**
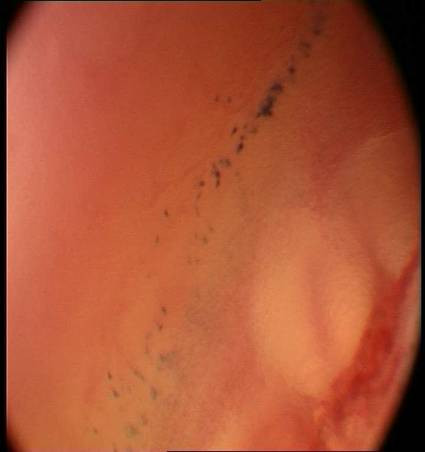
Mid sweep microendoscopic examination (x60) of buccal mucosa showing mucosal unruffling and spread of methylene blue stained nuclei.

**Figure 5:**
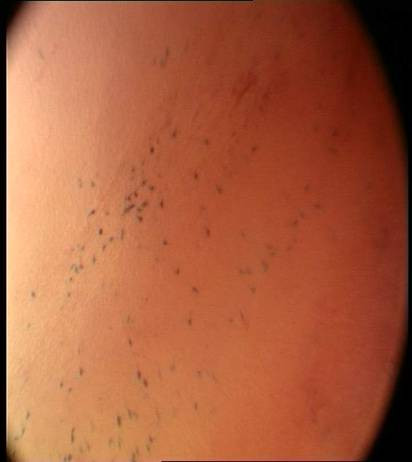
Post sweep microendoscopic examination (x60) of buccal mucosa showing mucosal unruffling and the even spread of methylene blue stained nuclei which gives the tissue a normal appearance.

At a microscopic level of tissue detail it can be seen that many epithelial surfaces are ruffled or concertinaed which distorts the image viewed and causes the impression of a high nuclear density per image field. This is particularly prevalent in areas of lax sub-mucosa such are the buccal mucosa, floor of the mouth, valleculae...etc. We have found that by spirally sweeping the endoscope across the field of view, the mucosa can be unruffled and that a truer image can be determined. This dynamic un-ruffling is seen in Figures 3, 4 &5.

**Figure 6:**
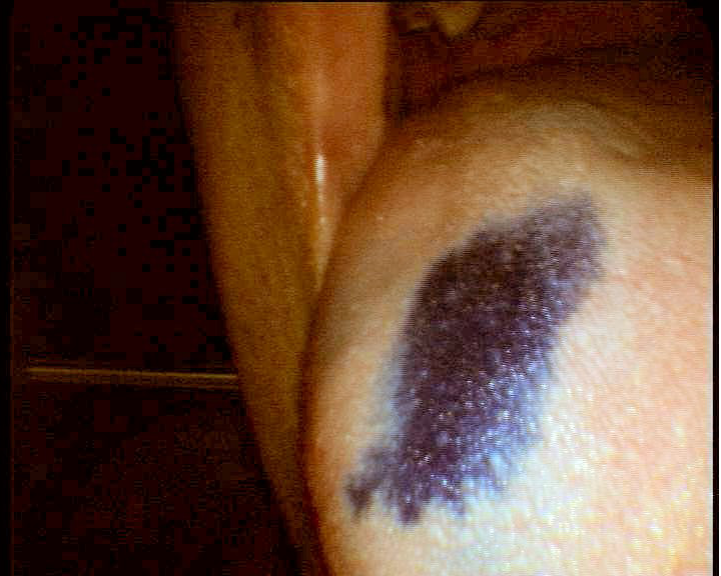
Methylene blue staining of the dorsum of the tongue prior to mucosal mircoendoscopic assessment.

**Figure 7:**
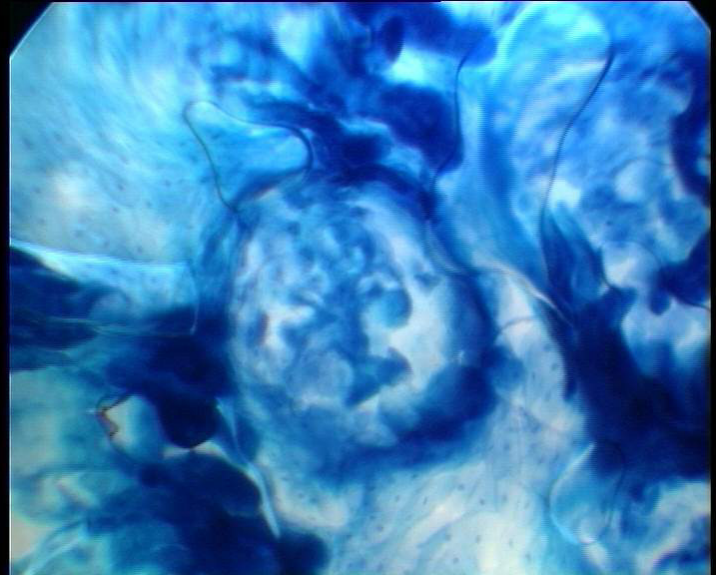
Microendoscopic assessment of the same area in "Figure 6" showing the surface view of a taste bud, with the nuclei and cell borders outlined by methylene blue dye. The mucosa is normal in appearance.

We used 0.1% Methylene blue as our vital staining agent; this was determined by using a dilution series of various stains (Evan's blue, Lugol, Toludine, Waterman and Methylene blue) on an area of normal mucosa with photo-documentation (Figures 6 &7). The ideal image (and corresponding dye dilution) was determined by two independent examiners, who assessed staining, cellular and nuclear detail.

**Figure 8:**
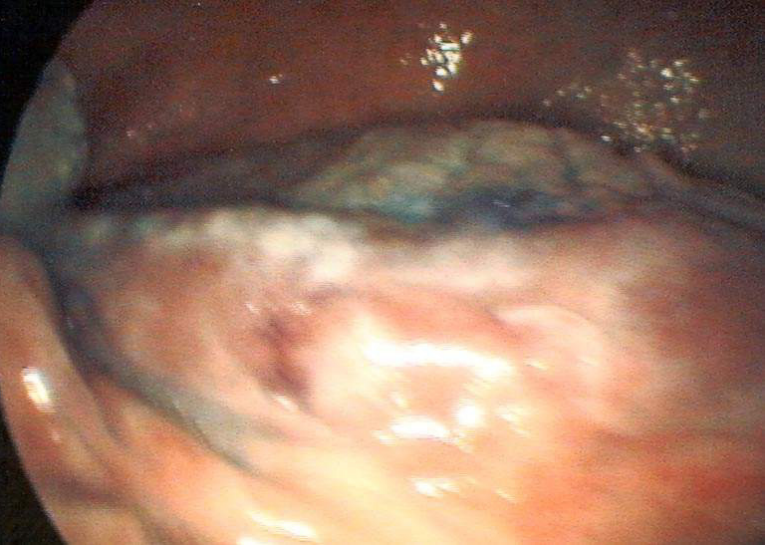
Clinical photograph of an edentulous patient presenting with a sore white patch in the floor of the mouth. This is prior to methylene blue staining.

**Figure 9:**
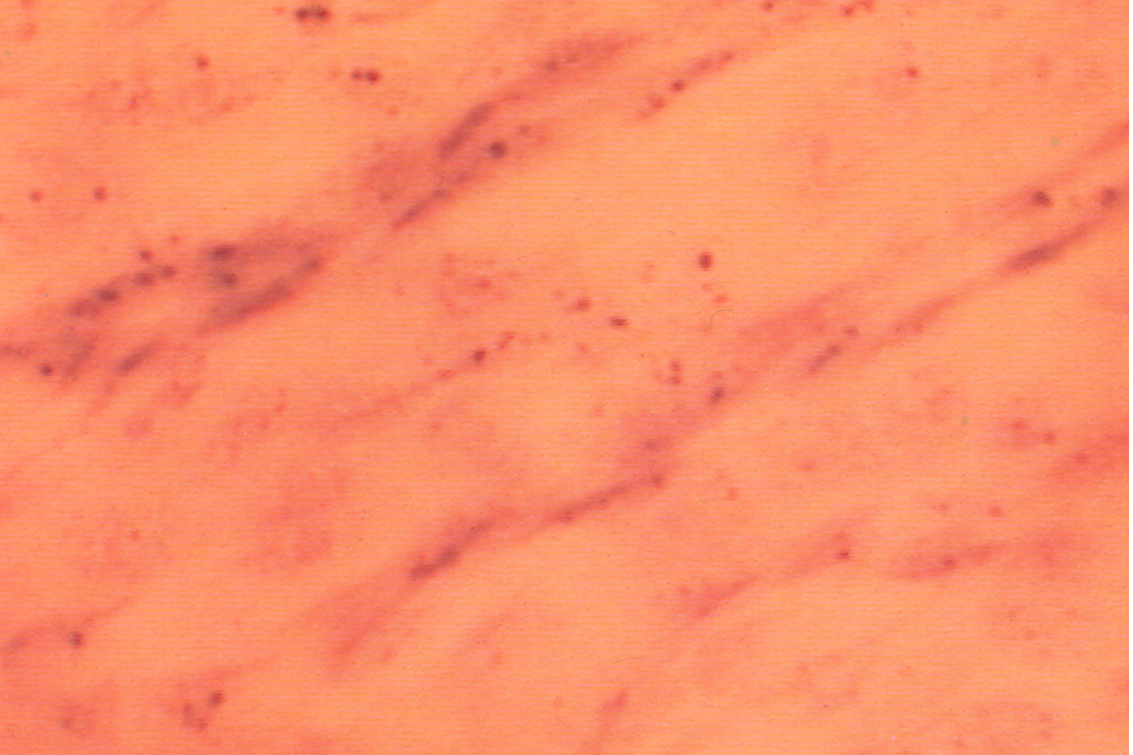
Microendoscopic image (x150) of the surface of the white patch in "Figure 8" showing the typical background appearance of keratosis with deeper nuclei visible as streaks and Civatte bodies (indicative of Lichen Planus).

**Figure 10:**
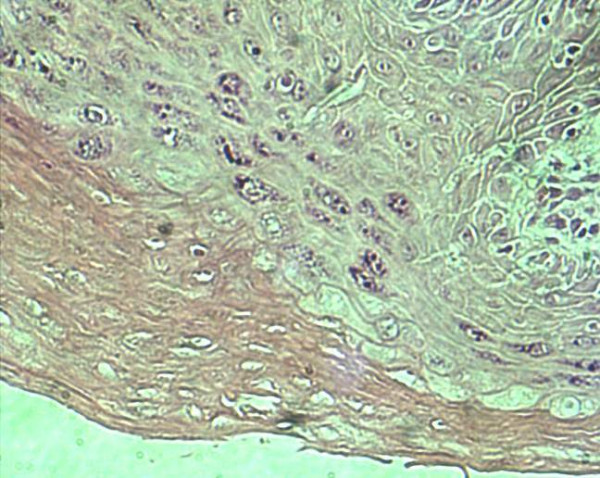
Histopathological image of a biopsy guided by the assessment in "Figure 9" showing keratosis with scattered nuclei (20–40 cell layers deep) and Civatte bodies at the basement membrane conducive with a diagnosis of Lichen Planus. This is at right angles (i.e. transverse section) to the plane of assessment in "Figure 9".

**Figure 11:**
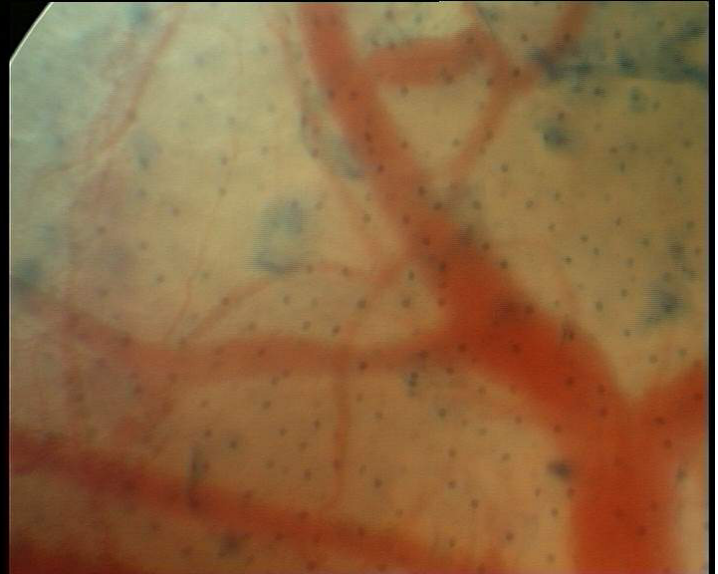
Microendoscopic view x60 showing a capillary network and nexus of blood vessels.

After suction clearance, the tip of the microendoscope is firmly applied to the pre-stained area of interest to obtain an occlusive contact and then moved for dynamic assessment of the lesion, its margins, the local tissue and the underlying mucosal vasculature and blood flow. The examination was found to be reproducible between operators. The depth of field was altered on the microendoscope to visualize the sub mucosal and sub-serosal capillary networks (Figures 8, 9, 10 &11).

The examination was performed from an area of normality across the margin of the lesion to the centre of the lesion to allow correlation with the normal histology of the area. The colour from the dye lasts 4–5 mins and gradually dissipates; further staining might be required for further assessment. The examination was performed at operation *in situ *and repeated on the excised *ex vivo *specimens.

### Statistical analysis

Inter-observer Kappa scores were calculated as well as sensitivity and specificity (comparing microendoscopy to gold standard paraffin section histology) using Chi-square testing. The cases were presented randomly to the examiners using a computer random number generator.

## Results

By using the above diagnostic criteria, the microendoscope was found to have sensitivities and specificities of 95.0% and 90.0%, respectively, in its ability to determine abnormal mucosa compared to formal gold standard histopathological parafin section examination (Table 1).

**Table 1:** Sensitivity and specificity measured against paraffin section histology

	**HISTOLOGICAL DIAGNOSIS**			
		**Malignant**	**Normal**	**Total**

**Microendoscopy DIAGNOSIS**	**Malignant**	19	2	**21**
	**Normal**	1	18	**19**
	**Total**	**20**	**20**	
**Sensitivity = TP/(TP+FN)**			**95.0%**	
**Specificity = TN/(TN+FP)**			**90.0%**	

Forty cases were examined by both observers with a Kappa statistic 0.85 (95% confidence interval of 0.687 to 1.013). The strength of agreement was considered to be 'very good' (Table 2).

**Table 2:** Interobserver reliability

Number of observed agreements	**37 **(92.50% of the observations)
Number of agreements expected by chance	**20.0 **(50.00% of the observations)
**Kappa = 0.850**	
**95% confidence interval**	0.687 to 1.013
The strength of agreement is considered to be	'**very good**'

We have found the procedure and results reproducible between operators given that the microendoscopic image is actually visible on the monitors for the clinician to see and that the image capture to storage media or printer can be correlated with later histopathological analysis. The team took six months to optomise the technique and become proficient. We found that very detailed correlation with histopathological parafin sections of the area was essential to speed the learning curve.

## Discussion

The active migration of cells towards the surface epithelium during maturational turn-over would tend to suggest that microendoscopic examination of the surface epithelium would be justified to detect underling mucosal pathology [1,2]. The potential pitfalls of the technique are that it is a new skill and that the equipment is still a relative expense; however skills can be learnt and costs can be reduced by the local manufacture of the various adaptors necessary to connect the microendoscope to existing camera systems (Laparoscopic, Cystoscopic, Otolaryngological).

In our optical diagnostics interest group it took nearly six months to establish the workable methodology and minimum diagnostic criteria we present in this paper. Despite this, the initial learning curve was high especially when ex-vivo specimens are used.

A fundamental knowledge of regional histology and pathology is necessary [3]. We feel there must be a high degree of cooperation between the surgeon and histopathologist. Perhaps initially correlating microendoscopic still or video footage with formal histology slides until confidence is gained. This correlative exercise should continue until a measure of proficiency is gained in topographical histopathologic interpretation. Local specificity and sensitivity in detecting areas of abnormality should be ascertained to develop skills of having a raised index of suspicion of when to biopsy.

The diagnostic criteria which were difficult to observe without a microscope and which differ from routine clinical examination were nuclear, cellular and tissue details suggesting abnormality (Table 3). For example, unstable mucosa will have high cell turnover and the amount of nuclear staining will be increased across all depths of the mucosa. Adverse microscopic predictors of abnormality at the cellular level (Table 4) would include the presence of a large nuclear to cytoplasmic ratio in cells together with evident bizarre shaped nuclear staining and the presence of numerous mitotic figures per field (two or more stained nucleoli per cell suggesting aneuoploidy); at the tissue level there is punctuate staining of cells and the heaping up of bizarre sheets of stained cells suggesting rapid cell turnover. There may even be pseudo-tissue borders in unexpected sites for example not at traditional squamo-columnar junction sites. Another adverse predictor of abnormality was more than 2 visible vessels per high power field suggesting high micro-vessel density (a feature common to many angiogenically active lesions).

**Table 3:** Diagnostic criteria assessed to determine abnormality

**Cellular level**	Cell: seen in longitudinal tissue plane rather than transverse/depth
	Cellular morphology, expected histology
	Nuclear staining pattern- orientation, size, shape, limits
	Nucleolar staining morphological pattern, orientation
**Tissue Level**	Cell-cell regularity
	Extracellular matrix- homogenous, heterogeneous
	Margin- discrete, blurred
	Underlying cells, micro-vessel density

**Table 4:** Adverse microscopic predictors

**Cellular level**	Large nuclear to cytoplasmic ratio
	Bizarre shaped nuclear staining
	The presence of numerous mitotic figures per field
	The presence of 2 or more stained nucleoli per cell suggesting aneuloploidy
**Tissue Level**	Punctate staining of cells
	Heaping up of stained cells suggesting rapid cell turnover
	Bizarre sheets of cells
	Distinct tissue borders in unexpected sites e.g. Not at traditional squamo-columnar junctions
	More than 2 visible vessel per high power field suggesting high micro-vessel density

It is not possible to determine the progression from dysplasia to carcinoma on the basis of the clinical finding. Such a progression of mucosal change cannot be detected because areas of suspected mucosal change may contain foci of varying degrees of dysplasia. Regular follow-up examinations are therefore essential for precancerous lesion and microendoscopically assisted assessment is ideally suited to this challenge.

The disadvantages of this technique are that the microendoscope does not provide direct three dimensional information concerning depth of invasion. However this may be ascertained by a series of 'MOHR'S'-like 2 dimensional analyses using the microendoscope. A degree of depth of field information may be obtained by varying the focus to allow visualization of underlying structures cells or blood vessels. Cellular detail can be determined up to tens of cell layers deep depending on illumination. Intra-wound interpretation using the microendoscope for assessing deep margins is difficult and requires a thorough understanding of the topographical histopathological appearance of the area especially when observing the oblique cuts made with excision. It also requires the surgeon to have an intimate knowledge of the histology of the area on which they are operating.

The technique of microendoscopy will no doubt be improved by advances in optical systems, illumination, recording and image processing. The microendoscope has a range of exciting applications in Head & Neck, Plastic and General Surgery [3,4]. The main advance with the endoscope is that we have more informed choice of the state of the *in situ *epithelial margin taken when excising squamous cell carcinoma by having better information about the tumour margin. This together with the ability of the endoscope to optically sample virtually the whole of the local mucosal area means that not only will we know where the margins are but will also be able to obtain biopsy samples from areas that will give the highest diagnostic yield. Combined with the novel vital dyes and antibody tagged immunologically targeted staining. Microendoscopy will advance the type of surgical margin we take from the standard clinically visible margin to that of a histopathological margin. In the near future we may even obtain molecular margin by antibody detected change and staining of cells expressing characteristics associated with malignant change or pre-malignant change. We suspect that the application of this technique will have major ramifications with regard to the clinical staging of Head & Neck tumours, with the 'newer' scopes allowing 'in-situ' estimations of depth of penetration (presently up to 5mm correlate Figures 9 and 10), vascular and lymphatic invasion. Hopefully the use of this technique should improve T staging assessment of the area and to some extent more prognostically significantly of the volume (via depth) of disease.

## Conclusion

Using the microendoscope we report our experience in the determination of surgical margins at operation and later comparison with frozen section and paraffin section margins "gold standard". We were able to obtain a sensitivity of 95% and a specificity of 90%. Inter-observer Kappa scores comparing the microendoscope with formal histological analysis of normal and abnormal mucosa were 0.85.

The advantage of this technique is that a large area of mucosa can be sampled and any histomorphological changes can be visualised in real time allowing the operator to make important informed decisions with regards the intra-operative resection margin at the time of the surgery.

Microendoscopy of the upper aero-digestive tract is still a novel technique which shows potential in the management of head and neck pathology with many related clinical applications. For the technique to be successful the surgeon, pathologist and histopathologist will need to be familiar with the subtleties of microendoscopy; this will require close collaboration among different specialties.

## Authors' contributions

TU designed the study, carried out the literature research, clinical study, statistical analysis and manuscript preparation

CF designed the study, carried out the literature research, clinical study and manuscript preparation

WJ designed the study, carried out the literature research, clinical study and manuscript preparation

ME designed the study, carried out the literature research, clinical study and manuscript preparation

SS carried out the literature research, manuscript preparation, and manuscript review

HS carried out the literature research, manuscript preparation, and manuscript review

AS carried out the manuscript editing and manuscript review

DA carried out the manuscript editing and manuscript review

LM designed the study, carried out the literature research and manuscript preparation

CH designed the study, carried out the literature research and manuscript preparation

PR designed the study, carried out the literature research and manuscript preparation

DH designed the study, carried out the literature research and manuscript preparation

AW designed the study, carried out the literature research, clinical study and manuscript preparation

## References

[B1] Silverman S, Gorsky M, Lozada F (1984). Oral leukoplakia and malignant transformation. A follow up study of 257 patients. Cancer.

[B2] Karabutul A, Reibel J, Therkildsen MH, Praetorius F, Nielsen HW, Dabelsteen E (1989). Observer variability in the histological assessment of oral pre-malignant lesions. J Oral Pathol.

[B3] Silverman S, Dillon WP (1982). Diagnosis in Oral cancer 3^rd ^London. Churchill Livingstone.

[B4] Clinel A, Oselladore M, Insacco E, Minucci D (1990). The accuracy of colposcopically directed biopsy in the diagnosis of cervical intraepithelial neoplasia. Eur J Gynaec Oncol.

[B5] Gynther G, Rozell B, Heimdahl A (2000). Direct oral microscopy and its value in diagnosing mucosal lesions. Oral surgery Oral medicine Oral pathology Aug.

[B6] Andrea M, Santos DiasO (1995). Contact endoscopy during micro-laryngeal surgery. A new technique for the endoscopic examination of the larynx. Ann Otol Rhinol Laryngol.

[B7] Andrea M, Dias O (2001). Contact endoscopy of the Upper Aerodigestive Tract.

